# Microbial Reduction of Fumonisin B1 by the New Isolate *Serratia marcescens* 329-2

**DOI:** 10.3390/toxins13090638

**Published:** 2021-09-10

**Authors:** Pisut Keawmanee, Chainarong Rattanakreetakul, Ratiya Pongpisutta

**Affiliations:** 1 Department of Plant Pathology, Faculty of Agriculture at Kamphaeng Saen, Kasetsart University, Nakhon Pathom 73140, Thailand; pisut.keaw@ku.th (P.K.); agrryp@ku.ac.th (R.P.); 2 Postharvest Technology Innovation Center, Ministry of Higher Education, Science, Research and Innovation, Bangkok 10400, Thailand

**Keywords:** fumonisin, mycotoxin reduction, *Serratia marcescens*

## Abstract

The mycotoxin fumonisin (FB) has become a major problem in maize products in southeastern Asia. Fumonisin can affect the health of humans and many animals. Fumonisin contamination can be reduced by detoxifying microbial enzyme. Screening of 95 potent natural sources resulted in 5.3% of samples yielding a total of five bacterial isolates that were a promising solution, reducing approximately 10.0–30.0% of fumonisin B1 (FB1). *Serratia marcescens*, one of the dominant degrading bacteria, was identified with Gram staining, 16S rRNA gene, and MALDI-TOF/TOF MS. Cell-free extract showed the highest fumonisin reduction rates, 30.3% in solution and 37.0% in maize. Crude proteins from bacterial cells were analyzed with a label-free quantification technique. The results showed that hydrolase enzymes and transferase enzymes that can cooperate in the fumonisin degradation process were highly expressed in comparison to their levels in a control. These studies have shown that *S. marcescens* 329-2 is a new potential bacterium for FB1 reduction, and the production of FB1-reducing enzymes should be further explored.

## 1. Introduction

Mycotoxins, which are secondary metabolites produced by fungi [1], cause serious problems to animal and human health. Plant pathogenic fungi are one of the fungal groups causing crop health problems. This damage has a direct effect on agricultural production and the economy [2]. Mycotoxins are accumulated during fungal colonization of plants by fungi before harvest. Fungi such as *Fusarium graminearum*, *F. verticillioides*, *F. proliferatum*, and sometimes *Aspergillus flavus* are present before harvest. Another group of fungi can occur after harvest, as reported for so-called storage fungi such as *Penicillium verrucosum* and *A. flavus* [3,4]

Fumonisins are mycotoxins that are mainly produced by *F. verticillioides* (Sacc.) Nirenberg (previously *F. moniliforme*, Sheldon) and *F. proliferatum* (Matsush.) Nirenberg [5]. To date, 28 structurally related fumonisin analogs have been identified. Three of the fumonisins B1, B2, and B3 occur abundantly [6]. Fumonisin B1 (FB1) is a highly toxic fumonisin analog that causes equine leukoencephalomalacia in horses, hepatocarcinogenesis in rats, and pulmonary edema in swine [7]. Maize products are primarily contaminated with fumonisins [8], which are related to starburst symptoms in maize [9,10]. In the Biomin world mycotoxin survey 2020, it was reported that fumonisins contaminated various commodities, especially maize. The contaminated maize was found at high levels of 96% in Asia, 70% in North America, and 71% in Europe. Some positive samples were reported to contain maximum concentrations of fumonisins at 30,872 ppb, 66,588 ppb, and 13,902 ppb, respectively [11].

Physical, chemical, and biological principles are used to develop strategies to eliminate fumonisin contamination in food and feed. Even so, physical, and chemical approaches have certain drawbacks in terms of costly instrumentation and nutritional losses. Biological detoxification using an enzyme technology is a promising strategy. Enzymes can reduce mycotoxin toxicity by transforming mycotoxins to less toxic metabolites. In some cases, the use of an enzyme can provide a practical approach to the nutrition in feed [12,13,14,15].

The first report of fumonisin microbial detoxification was given by Duvick et al. [16]. Microbes were isolated from moldy maize kernels and stalk tissue. They can grow with FB1 as their sole carbon source. They were Gram-negative bacteria identified as *Exophiala spinifera* and *Rhinocladiella atrovirens*. In 1999, Blackwell et al. [17] reported that the fungal species *E. spinifera* produces soluble extracellular esterase and can transform FB1 to hydrolyzed FB1, the amino polyol AP1, and free tricarballylic acid. The hydrolyzed FB1 has been demonstrated to have a greatly reduced toxicity compared to FB1 [18]. Benedetti et al. [19] reported that the bacterial strain NCB 1492, isolated from soil samples using an enrichment culture technique, can degrade FB1 as the sole carbon and nitrogen source in phosphate buffer. Sequences identified using 16S rDNA analysis were related to *Delftia/Comamonas*. Heinl et al. [20] investigated two genes involved in fumonisin degradation from *Sphingopyxis* sp. MTA144. The deesterification of FB1 to hydrolyzed FB1 was catalyzed by recombinant carboxylesterase in the same manner as the deamination of hydrolyzed FB1 in the presence of pyruvate and pyridoxal phosphate. In 2016, Masching et al. [21] noted that a commercial FUMzyme feed supplement that contains the fumonisin carboxylesterase FumD prevented changes in the sphinganine-to-sphingosin (Sa/So) ratio in turkeys and pigs. Hence, only a few microorganisms and enzymes have been successful in reducing FB1. The objectives of this study were to screen for fumonisin-degrading bacterial strains in natural sources and determine the proteomic profile of bacterial intracellular enzymes with a regard to FB1 reduction.

## 2. Results

### 2.1. Acclimatization and Isolation of Potential Fumonisin-Degrading Bacteria

Screening of bacteria for fumonisin degradation was performed through an acclimatization process. After acclimatization of natural sources such as maize, rice, soil, and fermented fluid with crude fumonisins, the degradation efficiency was determined. From the potential samples from 95 natural sources, we found five samples from which FB1-degrading bacteria were isolated. The FB1-degrading bacteria from natural sources were in approximately 5.3% of the natural source samples. Four were found in maize samples (isolates S2, 302-2, 329-2, and 412), and one was from fermented fluid (isolate P1) (Table 1). All bacteria were purified and collected for further study.

### 2.2. Fumonisin B1 Removal Activity by a Selected Bacterial Isolate and Determination of the Active Components

**Figure 1:**
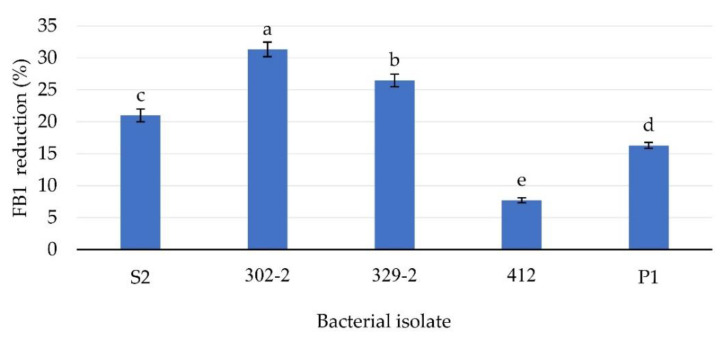
Percentage of FB1 reduction by all isolates after 24 h of incubation with the FB1 standard at 5 ppm. Different lowercase letters above the columns represent significant differences by ANOVA (*p* < 0.05), *n* = 5.

Each selected bacterial isolate was tested using PBS containing FB1 at 5 ppm and the FB1 degrading activities were observed. The reduction rates of these isolates ranged from 7.72% to 31.34% after 24 h of incubation. The highest FB1 reduction rate (31.34%) was exhibited by bacterial isolate 302-2, followed by isolate 329-2 at 26.48% (Figure 1).

**Figure 2:**
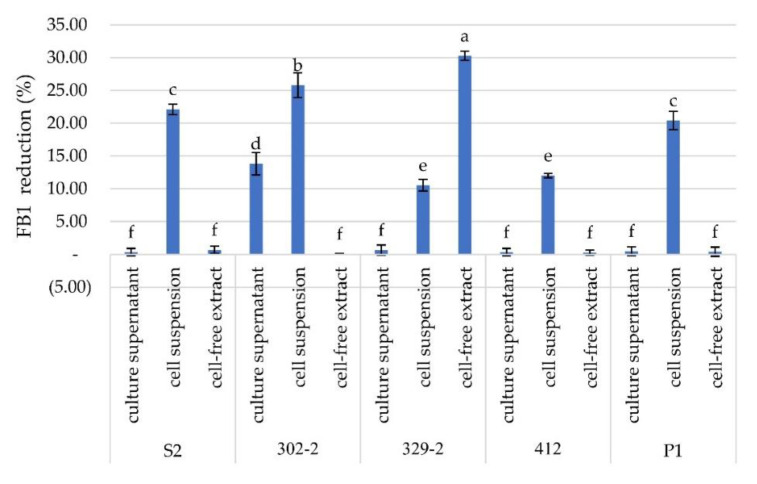
Percentage of FB1 reduction by culture supernatants, cell suspensions, and cell-free extracts in solution after 24 h of incubation with FB1 standard at 5 ppm. Different lowercase letters above the columns represent significant differences by ANOVA (*p* < 0.05), *n* = 5.

**Figure 3:**
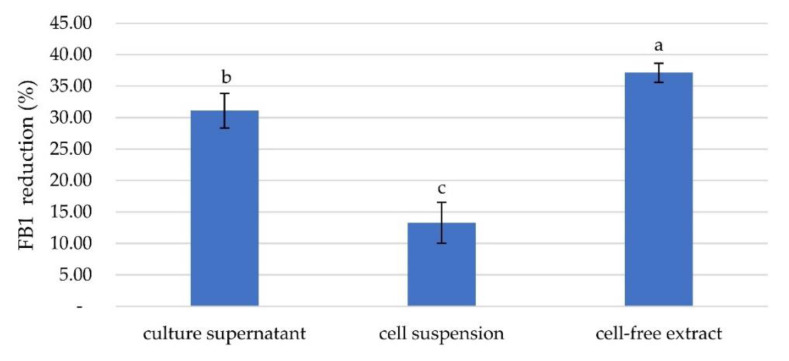
Percentage of FB1 reduction by the culture supernatant, cell suspension, and cell-free extract in ground maize after 24 h of incubation with the FB1 standard at 5 ppm. Different lowercase letters above the columns represent significant differences by ANOVA (*p* < 0.05), *n* = 5.

For further investigation, three bacterial preparations, the cell suspension, culture supernatant, and cell-free extract, were prepared from the five bacterial isolates. The efficiency of FB1 degradation was determined for each portion. The percent reduction ranged from 0–30.29%. The highest reduction occurred with treatment by isolate 329-2 cell-free extract, which resulted in a reduction rate of 30.29%, followed by reductions of 25.80% using the cell suspension of 302-2 and 22.13% using the cell suspension of S2. The reduction rate of 302-2 culture supernatant was 13.82%, while those of cell suspensions of 412 and 329-2 were 12.02% and 10.55%, respectively. Culture supernatants and cell-free extract of bacterial isolates S2, 412, and P1 showed no reduction; moreover, the culture supernatant of isolate 329-2 was effective. Each portion showed a different reduction rate. This result might be due to the active protein involved in FB1 reduction, which was contained in different bacterial fractions. (Figure 2). An in situ study showed that bacterial isolate 329-2 was the most capable of FB1 degradation. Then, further study of cell-free bacterial isolate 329-2 extract was performed in ground maize. The cell-free extract of 329-2 had the highest reduction rate at 37.00%, followed by the culture supernatant at 31.30% and by the cell suspension at 13.40% (Figure 3).

### 2.3. Bacterial Identification

**Figure 4:**
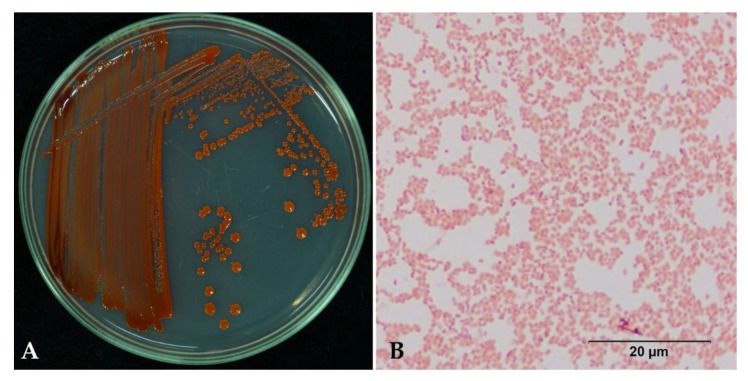
Microscopic and macroscopic examination of *Serratia marcescens* 329-2. (**A**) The colony morphology of 329-2 on nutrient glucose agar. (**B**) Rod-shaped cells observed by microscope.

Bacterial isolate 329-2 was isolated from maize and formed red pigmentation on round colonies and an entire margin on NGA after 48 h of incubation under aerobic conditions. The isolate was a rod-shaped and Gram-negative bacterium (Figure 4).

**Figure 5:**
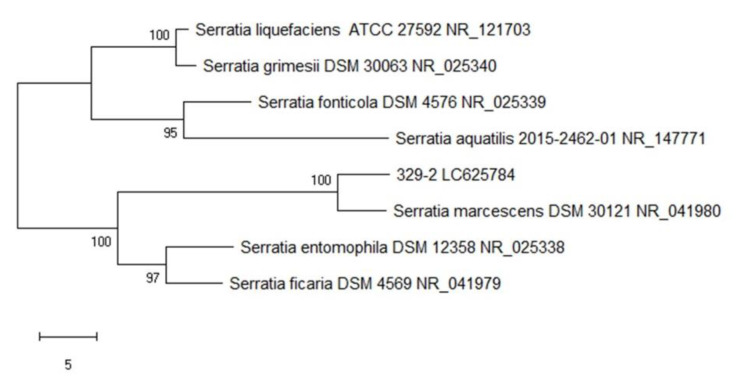
Phylogenetic tree based on 16S rRNA gene sequences of isolate 329-2 and related taxa.

The relationships of isolate 329-2 and other closely related bacterial species are shown in Figure 5. Isolate 329-2 appears to be closely related to *Serratia marcescens*. Furthermore, a BLAST search at the NCBI indicated that the 16S rDNA sequence of isolate 329-2 was most similar to that of *Serratia marcescens* DSM 30121 (accession number: NR_041980). The 16S rDNA sequence of isolate 329-2 has been deposited in the GenBank database under accession number LC625784.

Matrix-assisted laser desorption/ionization-time of flight mass spectrometry (MALDI-TOF/TOF MS) showed that isolate 329-2 belonged to *S. marcescens*, and the highest score (2.361) was for *S. marcescens* DSM 12481 (Table 2).

### 2.4. Proteins Expression during Fumonisin B1 Reduction by S. marcescens 329-2

**Figure 6:**
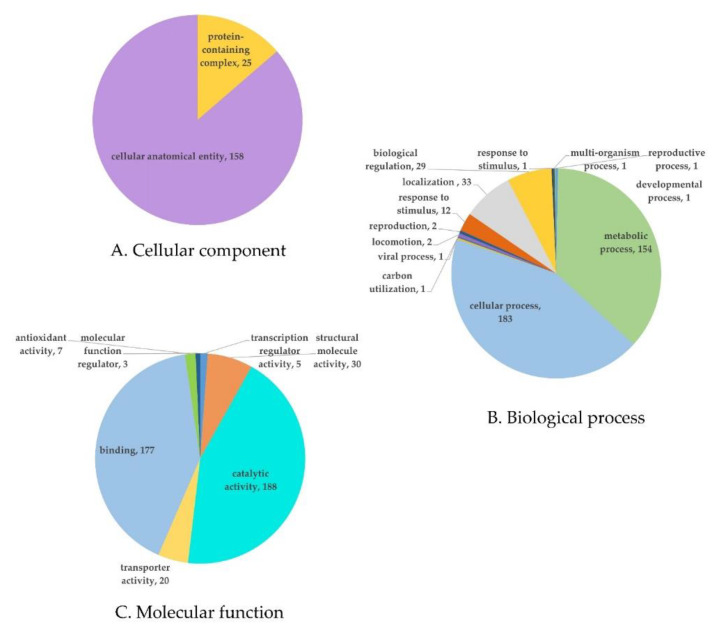
Gene ontology (GO) classifications of the differentially expressed proteins during fumonisin degradation by *S*. *marcescens* 329-2.

The protein expression results identified 461 differentially expressed proteins with *p* < 0.05, and of these proteins (Supplementary Tables S1 and S2), 159 showed upregulated expression and 25 showed downregulated expression in the treatment group. To evaluate the expression of protein functions, we annotated these proteins based on the gene ontology (GO) databases. The identified level 2 GO terms (related to cellular components, molecular functions, and biological processes) associated with the differentially expressed proteins are shown in Figure 6. Gene annotation of the expressed proteins showed their relationship to cellular components for 183 proteins, biological processes for 421 proteins, and molecular functions for 430 proteins. Major concerns exist regarding the biological process in which they are activated, and the proteins involved in cellular process (183) and metabolic process (154). In the category of molecular function, the majority of proteins were related to catalytic activity (188 proteins) and protein binding (177 proteins) (Figure 6).

**Figure 7:**
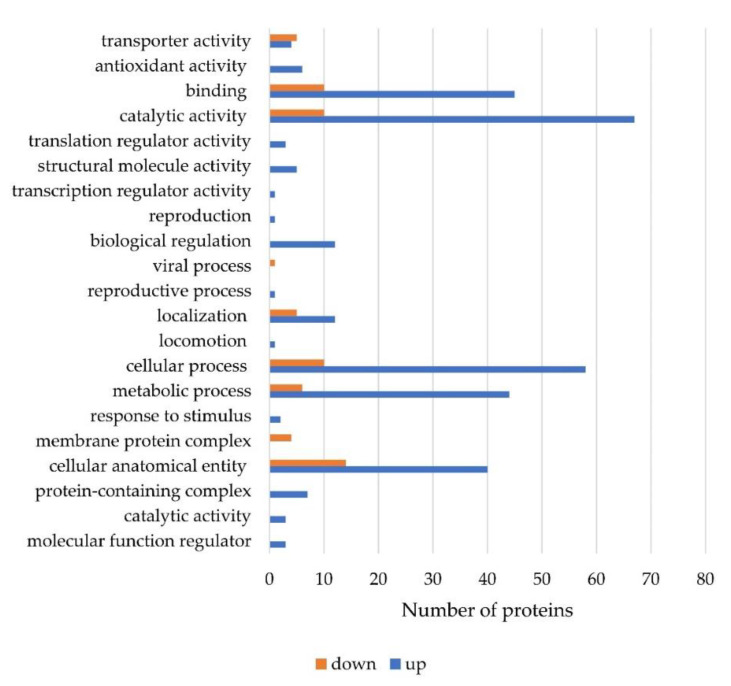
Comparison of protein upregulation and downregulation during fumonisin degradation by *S. marcescens* 329-2.

The highly matching proteins upregulated in FB1 degradation were categorized into catalytic activity, binding protein function, and metabolic process under in molecular function (Figure 7). The protein expression data are shown in Table 3 and Table 4, with the fold change values in expression relative to the noninduced protein levels from *S. marcescens*. The upregulated proteins in Table 3 indicate the proteins that were highly related to FB1 degradation by *S. marcescens*. The details of each protein were compared within the UniProt database. This indicates the function related to the protein degradation of FB1. In relation to FB1, proteins involved in the cell catalysis process include hydrolase proteins and aminotransferase proteins. The upregulated hydrolase function was evidenced by entries A0A6N3ZRH0 (fumarylacetoacetate hydrolase family protein, 4.91), A0A656VL53 (alpha/beta hydrolase, 4.90), A0A6I4GZS8 (hydrolase, 3.80), and A0A6M5I193 (MBL fold metallo-hydrolase, 3.64), and the transferase enzymes that can activate chemical groups in FB1 included A0A6H1E4N5 (Acetylornithine/succinyl-diaminopimelate aminotransferase, 4.1), Q6MXC8 (methyl transferase, 3.94), A0A656V5R8 (5-methyltetrahydropteroyltriglutamate-homocysteine methyltransferase, 3.46), and A0A0U6KIH4 (GTP cyclohydrolase, 3.34). Other proteins included the isomerase A0A0G8B4P9 (peptidyl-prolyl cis-trans isomerase, 3.58), the protein synthesis protein A0A6M5HVT2 (4-hydroxy-tetrahydrodipicolinate synthase, 5.33), and the cell metabolism protein V5YV29 (maltodextrin-binding protein, 6.89). ABC transporter, cellular process, transcription, nucleic acid proteins were also induced, which means that cellular processes were also activated for the degradation response.

Proteins that were downregulated (Table 4), which means that the proteins that were highly present in the control group, were mostly related to cellular anatomical entities and cellular processes and included proteins such as ATP synthase, glyceraldehyde-3-phosphate dehydrogenase, fructose-1,6-bisphosphatase class, and outer membrane proteins.

## 3. Discussion

FB1 is one of the most important mycotoxins produced by several species of *Fusarium*, mainly *F. verticillioides* or *F. proliferatum*, which frequently occur in maize kernels and affect grain quality [22,23]. FB1 is a potential natural contaminating toxin. The fumonisin-contaminated products increase yearly with global warming [24]. The degradation of fumonisins is a concern because it causes contamination of feed products. To decrease the severity of contamination with fumonisin-producing fungi in the field before harvest, biocontrol agents against fumonisin-producing fungi have been studied, such as lactic acid bacteria from corn silage [25], *Pediococcus pentosaceus* (L006) isolated from maize leaves [26], and *Lactobacillus plantarum* MYS6, a probiotic bacterium [27]. In an early report, Becker et al. [28] treated 50 to 1000 µM fumonisin with human intestinal bacteria. The results showed no effect on fumonisin decrease, bacterial growth, or metabolic substances. From our study, only 5.3% of natural tested samples (n = 95) showed FB1 degradation with the acclimatized method. Most of the potential samples were from maize and fermented fluid. Within this study, five bacterial isolates caused reductions of 8 to 32%. Although fumonisins are a highly important mycotoxin in maize samples worldwide, only some microorganisms, such as *Exophiala spinifera* isolate 2141.10, *Rhinocladiella atrovirens* and the Gram-negative bacterium 2412.1 isolated from maize [16,17], the *Delftia/Comamonas* group isolated from soil [19], and the microbial consortium SAAS79 isolated from spent mushroom compost [12], have been reported to effectively degrade FB1 at the post-harvest stage. In this study, the cell-free extract showed the highest fumonisin reducing rate, at 40% in solution and 30% in maize. The FB1 reduction factor may be a crude enzyme from inside bacterial cells [12,29].

In this study, we identified a bacterial isolate based on Gram staining, the 16S rRNA gene, and MALDI-TOF MS. Regarding the macroscopic characteristics, bacterial isolate 329-2 formed red pigment, round colonies, and an entire margin. The bacterium was Gram-negative, straight rod–shaped and 0.6–0.8 µM in diameter, similar to *S. marcescens* [30,31].

The 16S rRNA gene, used to investigate bacterial phylogeny and taxonomy, is the most frequent housekeeping genetic marker and is more reliable than other genes for various reasons. First, the 16S rRNA gene is present in all bacteria, typically as part of a multigene family or operon. Second, the 16S rRNA gene is a more accurate measure over time and is not altered, implying that random sequence changes are a more accurate measure of time. Finally, the 16S rRNA gene is large enough for informatics purposes [32,33,34]. The strain here whose 16S rRNA gene was sequenced was identified as a *S. marcescens* strain closely related to *S. marcescens* DSM 30121 (accession number: NR_041980). The 16S rRNA gene was also successfully used for *Serratia* species identification [35,36].

MALDI-TOF MS is an efficient high-throughput technology for identifying and evaluating proteins [37,38]. MALDI-TOF MS was successful for the identification of proteins from whole bacterial cells from various sources [39,40,41] and from *S. marcescens* [42,43]. In this study, the MALDI-TOF/TOF MS results showed isolate 329-2 belonging to *S. marcescens*. The highest score for matching was 2.361 for *S. marcescens* DSM 12481, indicating highly probable species identification [44]. Based on Gram staining, 16S rRNA gene sequence analysis, and MALDI-TOF MS, we conclude that bacterial isolate 329-2 was *S. marcescens*.

*S. marcescens* has been reported to be a potential biocontrol agent for plant pathogens causing several diseases such as damping-off disease in cyclamen caused by *Rhizoctonia solani* [45], damping-off disease in cucumber caused by *Phytophthora capsici* [46], and blast disease caused by *Pyricularia oryzae* in rice [47]. Moreover, *S. marcescens* has been demonstrated to be a plant growth-promoting agent inducing systemic resistance in cucumber against *Fusarium* wilt disease caused by *F. oxysporum* [48] and it can induce systemic resistance, enhanced salinity tolerance, and inhibit *F. graminearum* infection in wheat [49]. Guo et al. [50] reported that *S. marcescens* inhibited the germination of *F. proliferatum* and suppressed fumonisins accumulation in an in vitro study. However, the use of *S. marcescens* as a biocontrol agent was still concerned about a human opportunistic pathogen. In our study, we focused on intracellular enzymes without bacterial living cells. Red-pigmented *S. marcescens* 329-2 was used for a potential new enzyme from bacterial sources. It was not similar to the strain described by Carbonell et al. [51], as the non-pigmented strain of *S. marcescens* was mostly a human opportunistic pathogen.

To understand the proteins related to the degradation process, bacterial cells were analyzed with label-free techniques. Label-free MS-based quantitative proteomic analysis was attempted to further characterize fumonisin degradation. Many abundant proteins identified and quantified in the degradation process were upregulated, as shown in Table 3, or downregulated, as shown in Table 4. The major points of interest were indicated in a previous report by Blackwell, Gilliam, Savard, Miller and Duvick [17] on a soluble extracellular esterase from *E. spinifera* isolate 2141.10, which transformed fumonisin B1 to the amino polyol AP1 and free tricarballylic acid. Moreover, two genes encoding a carboxylesterases (*fumD*) and aminotransferases (*fumI*) for fumonisin degradation by esterification and hydrolysis were described from the bacterium *Sphingopyxis* sp. [20,52].

The label-free quantification data showed upregulated esterase and transferase proteins. Potential proteins associated with fumonisin degradation, including hydrolases and transferases, were examined. Potential proteins associated with fumonisin degradation included alpha/beta hydrolase (A0A656VL53) and acetylornithine/succinyldiaminopimelate aminotransferase (A0A6H1E4N5), with fold changes of 4.90 and 4.10, respectively.

Alpha/beta hydrolases are hydrolase families. Hydrolases are a group of enzymes that act as biochemical catalysts. A hydrolase is an enzyme that catalyzes hydrolysis of C-O, C-N, C-C, and phosphoric anhydride bonds. The enzymes use H_2_O to break a chemical bond, which typically results in the degradation of a larger molecule into smaller molecules. Hydrolases are classified as EC 3 enzymes. One common example of hydrolase enzymes is esterases, which include enzymes such as lipases, phosphatases, glycosidases, peptidases, and nucleosidases [53,54]. Montella et al. [55] reported that esterases hydrolyze ester bonds, which are present in a wide range of insecticides, including fumonisin. Fumonisin B1 esterase (EC 3.1.1.87) [20]. Later, they named the gene encoding carboxylesterase activity *fumD*. We assume that alpha/beta hydrolase may be involved in FB1 conversion to HPB1 and tricarboxylic acids.

Transferases, an enzyme class, can transfer various chemical groups from one compound to another. The enzymes work with functional groups such as the amino group (-NH_2_) (transferred from amino acids to keto groups in the case of transaminase), phosphate, methyl (-CH_3_), and sulfur-containing groups. The enzymes may react with one end of fumonisin (-CHNH_2_CH_3_). One of the enzymes identified was acetylornithine/succinyldiaminopimelate aminotransferase, which is related to the -CHNH_2_CH_3_ end of the fumonisin structure.

The results revealed that the degradation activity of *S. marcescens* was related to upregulated hydrolase and transferase enzymes. The specific enzymes of *S. marcescens* can decrease FB1 abundance, but they may show different specificities for the structure of fumonisin than the carboxylesterase and aminotransferase from *Sphingopyxis* sp. that have been marketed as FUMzyme.

## 4. Conclusions

In this study, we isolated and identified the bacterium *S. marcescens* 329-2 from maize with high FB1 reduction activity. This is the first report of *S. marcescens* reducing FB1. The FB1 reduction by the cell-free extract of *S. marcescens* 329-2 was more effective than that by the culture supernatant and cell suspension in FB1 solution and maize. We also found hydrolase and transferase with upregulated expression in bacterial cells, which may indicate potential for the development of FB1-reducing enzymes.

## 5. Materials and Methods

### 5.1. Acclimatization and Isolation of Potential Fumonisin-Degrading Bacteria

#### 5.1.1. Acclimatization of Bacteria from Natural Resources

Natural sources such as maize (starburst symptom and fumonisin-contaminated samples), rice (bakanae disease samples), soil, and fermented fluid were collected randomly from the continuous production area in Thailand. Five grams or milliliters of sample was added to 50 mL of nutrient glucose broth (NGB, 3 g of beef extract, 5 g of peptone, and 5 g of glucose) and incubated for 24 h followed by transfer of 1 mL into 20 mL of NGB with crude fumonisins (total fumonisins B1, B2, and B3) at 3 ppm, which refer to average level of positive sample of maize contaminated fumonisins in Asia [11]. The crude fumonisins were prepared by the inoculation of ground maize with *F. verticillioides* (fumonisins-producing strain). After 45 d, the crude fumonisins were extracted with 70% methanol and filtrated samples were diluted 1:20 with distilled water before taking into ELISA assay (AgraQuant^®^ Total Fumonisin Assay, Romer Lab^®^, Singapore), range 0.25–5.0 ppm with LOD = 0.20 ppm and LOQ = 0.25 ppm. Two hundred microliters of conjugation solution were mixed with 100 μL of each standard or sample. One hundred microliters of the mixture were transferred into the reaction wells and incubated for 10 min. The reaction wells were washed 5 times with distilled water and added with 100 μL of substrate solution. After 5 min of incubation at RT, one hundred microliters of stop solution were added into the mixture. Absorbance was measured by a microplate reader using a 450 nm filter (Tecan, Hombrechtikon, Switzerland). The data was interpreted by the AQ FUM form provided by the company.

#### 5.1.2. Isolation of Potential Fumonisin-Degrading Bacteria

The acclimatized samples were incubated at room temperature on shaker in the dark for 15 d. One hundred microliters of bacterial suspension was prepared by using agar spread on nutrient glucose agar (NGA) plates. The plates were incubated with alternating periods of 12 h darkness/light at 25 ± 2 °C for 24 h. A single colony of the withstand bacteria was cross streaked on NGA three times. The pure culture was used for analysis of reduced FB1 activity.

### 5.2. Fumonisin B1 Removal Activity by a Selected Bacterial Isolate

Bacteria were identified based on the removal of FB1 as described by Niderkorn, Morgavi, Pujos, Tissandier and Boudra [25] with some modifications. Overnight culture of a bacterial pellet was adjusted to 0.2 OD using PBS, pH 7 (phosphate buffer solution; 8 g of NaCl, 0.2 g of KCl, 0.2 g of KH_2_PO_4_, and 1.44 g of Na_2_HPO_4_). One hundred microliters of bacterial suspension was mixed with FB1 solution (Biopure, Tulln, Austria) to final concentration of 5 ppm in a 1000 µL reaction. The concentration was set at 5 ppm according to the European Union maximum limits for fumonisins B1 and B2 established in the complementary and complete feeding stuffs for pigs, horses, rabbits, and pet animals [56]. The high FB1 concentration was set to strengthen the screening of potential selected bacterial isolates in a short period of 24 h. A positive control containing only FB1 in PBS and a negative control containing only a bacterial suspension in PBS were used. All tubes were incubated at 37 °C in the dark for 24 h. After incubation, the samples were determined the FB1 concentration with ELISA assay (AgraQuant^®^ Total Fumonisin Assay, Romer Lab^®^, Singapore), range 0.25–5.0 ppm with LOD = 0.20 ppm and LOQ = 0.25 ppm, followed topic 5.1.1. The rate of FB1 degradation was calculated using the following formula: (concentration of FB1 control-concentration of FB1 residual)/concentration of FB1 control × 100%.

### 5.3. Determination of the Active Components

The bacterial culture was separated into culture supernatant, cell suspension, and cell-free extract fractions. All components were used to screen for fumonisin-degrading activity according to Wang et al. [57]. The culture supernatant and cell suspension were obtained by centrifugation at 10,000 rpm and 4 °C for 15 min. Then, the culture supernatant was filtered through 0.22 μm sterile cellulose acetate filters. The cell pellet was washed twice with PBS, pH 7, before being suspended again in the same buffer. Cell-free extract was prepared by disintegrating (5 s on/off) the cell suspension using a sonicator for 30 min in an ice bath. After that, the cell debris was removed by centrifugation at 13,000 rpm and 4 °C for 15 min. To obtain cell-free extract, the supernatant was filtered through 0.22 μm sterile cellulose acetate filters.

FB1 removal activity was determined as follows: 250 µL of FB1 (10 µg/mL) was mixed with 250 µL of the culture supernatant, cell suspension, or cell extract. All reaction systems were placed on a rotary shaker for 24 h.

To determine the potential of active components in a realistic matrix, we used 20 g of 5 ppm spiked ground maize in a 50 mL laboratory bottle. Then, five hundred microliters of the culture supernatant, cell suspension, or cell-free extract was dropped in the middle of the ground maize and incubated for 24 h at RT.

Each sample was extracted according to the ELISA kit instructions. The result was compared to that of the controls without active components.

### 5.4. Bacterial Identification

Gram staining, 16S rRNA gene sequence analysis, and MALDI-TOF MS were used for bacterial identification. One loop of an overnight culture was smeared on a slide for Gram staining following the method reported by Davies et al. [58]. The GeneJET Genomic DNA Purification Kit (Thermo Scientific, Vilnius, Lithuania) was used to extract genomic DNA according to the manufacturer’s instructions. The 16S rRNA gene was amplified by polymerase chain reaction (PCR) using the primers 27F (AGAGTTTGATCCTGGCTCAG) and 1492R (GGTTACCTTGTTACGACTT) [59,60]. PCR amplification was performed by a T Gradient (Biometra, Goettingen, Germany). Amplification conditions consisted of pre-denaturation at 94 °C for 3 min followed by 35 cycles of 94 °C for 1 min, 56 °C for 30 s, and 72 °C for 1 min, with final extension at 72 °C for 10 min. PCR products were confirmed using agarose gel electrophoresis (1X agarose in TBE buffer). The PCR products were purified and sequenced by Sanger sequencing (Apical Scientific, Selangor, Malaysia). Nucleotide sequence comparisons were performed using the National Center for Biotechnology Information (NCBI) database (http://www.ncbi.nlm.nih.gov/BLAST/, accessed date 23 March 2021). Similar 16S rDNA sequences were downloaded from GenBank and manually reviewed, after which all the sequences were aligned, and a phylogenetic tree was constructed using neighbor joining by MEGA X [61].

Bacteria were transferred to NGA and incubated at room temperature for 24 h before analysis. MALDI-TOF MS was performed by the Salaya Central Instrument Facility, Mahidol University, Nakhon Pathom, using an Autoflex MALDI-TOF mass spectrometer (Bruker Daltonics, Bremen, Germany). The standard Bruker interpretive criteria were applied as follows: unreliable identification (score 0.000–1.699); probable genus identification (score 1.700–1.999); secure genus and probable species identification (score 2.000–2.299); and highly probable species identification (score 2.300–3.000) [44].

### 5.5. Proteins Expression during Fumonisin B1 Reduction by S. marcescens 329-2

A bacterial pellet was harvested after 3 days of incubation on NGB, washed with PBS and centrifuged at 10,000 rpm for 15 min. The bacterial pellet was incubated in 3 ppm FB1 solution, and a control was inoculated in PBS without FB1. The test condition was set for 7 days to ensure the protein expression during FB1 reduction.

The cell pellet was trypsin-digested following protocol. The cell pellet was washed twice in 100 µL of PBS. The cell pellet was resuspended twice in a lysis buffer (10% sodium deoxycholate (Tokyo Chemical Industry, Tokyo, Japan), 10 mM Tris (2-carboxyethyl) phosphine hydrochloride, (Sigma-Aldrich, St. Louis, MO, USA), 40 mM 2-chloroacetamide (Sigma-Aldrich, St. Louis, MO, USA), and 50 mM phosphate buffer, pH 8.0), boiled at 95 °C for 10 min, and sonicated for 15 min. Cell debris was pelleted by centrifugation at 10,000 rpm for 5 min, and the clarified lysate was transferred into a new tube. The lysate was diluted 1:10 for trypsin digestion using Trypsin Gold, mass spectrometry grade (Promega, Madison, WIUSA) at an enzyme/substrate ratio of 1:50, and digestion was performed overnight at 37 °C. The digested sample was acidified to a final concentration of trifluoroacetic acid (Sigma-Aldrich, St. Louis, MO, USA) at 0.5%, and sodium deoxycholate was extracted by adding an equal volume of ethyl acetate and vigorous shaking. The organic phase was removed after centrifugation at 10,000 rpm for 5 min. The aqueous solution was transferred to a new tube and submitted to lyophilization. Label-free quantification and data analysis were performed by the Salaya Central Instrument Facility, Mahidol University, Nakhon Pathom, with NanoLC (Ultimate 3000, Thermo Scientific, Vienna, Austria) using an Acclaim PepMap RSLC C18 column (75 µm × 150 mm, Thermo Scientific, Vienna, Austria). The mobile phases were 2% (*v/v*) acetonitrile with 0.1% (*v/v*) formic acid (phase A) and 80% (*v*/*v*) acetonitrile with 0.1% (*v*/*v*) formic acid (phase B). The linear gradient elution was as follows: 0–5 min, 3% B; 5–45 min, 3–45% B; 45–50 min, 90% B; and 50–60 min, 3% B. The masses of the peptides were determined using a Sciex Triple TOF 6600+ instrument (AB Sciex, Framingham, MA, USA).

## Figures and Tables

**Table 1:** Number of samples collected from various natural sources.

Natural Source	Number of Screened Samples	Potential Degrading Samples
Maize	37	4
Rice	12	0
Soil	8	0
Fermented fluid	38	1
Total	95	5

**Table 2:** MALDI-TOF/TOF MS analysis of *Serratia marcescens* 329-2.

Rank	Quality	Matched Pattern	Score
1	+++	*Serratia marcescens* DSM 12481 DSM	2.361
2	+++	*Serratia marcescens* DSM 12485 DSM	2.345
3	+++	*Serratia marcescens* 13103_1 CHB	2.319
4	+++	*Serratia marcescens* subsp. *marcescens* DSM 30121T DSM	2.306
5	++	*Serratia marcescens* subsp. *sakuensis* CIP 107489T HAM	2.240
6	++	*Serratia ureilytica* DSM 16952T DSM	2.155
7	++	*Serratia marcescens* DSM 30122 DSM	2.147
8	++	*Serratia marcescens* (PX) 24086109 MLD	2.062
9	++	*Serratia marcescens* DSM 12483 DSM	2.039
10	+	*Serratia entomophila* DSM 12358T DSM	1.942

**Table 3:** Identification of the upregulated proteins (>3-fold change) in FB1-treated *S. marcescens* 329-2 compared with the control group.

Entry	Protein Names	Gene Names	Fold Change
A0A6G9UZ48	ABC transporter substrate-binding protein	HCG50_10660	8.20
A0A6N0D898	Porin OmpC	*ompC*	7.94
A0A3E2ENK9	Amino acid ABC transporter substrate-binding protein	*gltI*	7.12
V5YV29	Maltodextrin-binding protein	*malE*	6.89
A0A080V044	Universal stress protein	*uspA*	5.42
A0A6M5HVT2	4-hydroxy-tetrahydrodipicolinate synthase	*dapA*	5.33
A0A6G9UQE2	ABC transporter substrate-binding protein	HCG50_08880	5.33
A0A1Q4NZ53	Superoxide dismutase	BHU62_14220	4.99
A0A6G9UU24	Phosphate-binding protein PstS	*pstS*	4.98
A0A6N3ZRH0	Fumarylacetoacetate hydrolase family protein	G3M84_1332	4.91
A0A656VL53	Alpha/beta hydrolase	AB868_03825	4.90
A0A6M5HYD0	Phenylacetate-CoA oxygenase/reductase subunit PaaK	*paaK*	4.78
A0A3E2EF40	Malate dehydrogenase	*mdh*	4.68
A0A6N0D0Q5	Neutral metalloproteinase	F0335_18215	4.49
A0A086FBX8	Transcription termination/antitermination protein NusG	*nusG*	4.39
A0A2V4FJ05	Peptide deformylase	*def*	4.35
V5YU98	Extracellular solute-binding protein	E4655_11925	4.27
A0A5Q8BY15	YtfJ family protein	EGJ31_19890	4.20
A0A1Q5WAZ4	Oligopeptide ABC transporter substrate-binding protein OppA	A8A12_03045	4.18
A0A6H1E4N5	Acetylornithine/succinyldiaminopimelate aminotransferase	*argD*	4.10
A0A6N3ZYZ9	2,3-diphosphoglycerate-dependent phosphoglycerate mutase	*gpmA*	4.06
A0A0P0Q8S3	ABC transporter substrate-binding protein	AR325_02675	4.06
A0A6N0CVJ7	Superoxide dismutase	*sodB*	4.03
A0A5Q8C0J8	Organic hydroperoxide resistance protein	EGJ31_14360	4.02
V5YUS0	Periplasmic serine endoprotease DegP-like	*degQ*	4.01
Q6MXC8	Methyltransferase	SMR0272	3.94
A0A2S4XAJ8	Surface composition regulator	*glgS*	3.89
A0A1Q5WEW3	Antibiotic biosynthesis monooxygenase	A8A12_06980	3.87
A0A6I4GZS8	Hydrolase	GMA22_24835	3.80
A0A221FKL4	UPF0234 protein BVG93_01845	BVG93_01845	3.75
A0A6H3S2C0	ATP-dependent protease subunit HslV	*hslV*	3.71
A0A6M5I193	MBL fold metallo-hydrolase	HMI62_20840	3.64
A0A6N0DB57	Protein deglycase HchA	*hchA*	3.62
A0A2V4G7I4	Amino acid ABC transporter substrate-binding protein	*glnH*	3.62
A0A5C7CH16	VOC family protein	FOT62_15570	3.58
A0A0G8B4P9	Peptidyl-prolyl cis-trans isomerase	*fkpA*	3.58
A0A656VU86	Long-chain fatty acid transport protein	AB868_00683	3.55
A0A2S4 × 857	Histidine ABC transporter substrate-binding protein HisJ	*hisJ*	3.47
A0A656V5R8	5-methyltetrahydropteroyltriglutamate--homocysteine methyltransferase	*metE*	3.46
A0A0G8BFE1	Cystine ABC transporter substrate-binding protein	*tcyJ*	3.43
A0A1C3HIZ5	Aconitate hydratase B	*acnB*	3.35
A0A0U6KIH4	GTP cyclohydrolase 1	*folE*	3.34
A0A6N0CW48	Branched-chain amino acid ABC transporter substrate-binding protein	F0335_15805	3.32
A0A6N3ZXZ8	ABC transporter substrate-binding protein	G3M84_09620	3.32
A0A6G8TTH4	Autoinducer 2-binding protein LsrB	G5643_21680	3.29
A0A656VPU2	Uncharacterized protein	AB868_00798	3.26
A0A1C3HHX7	Nitrogen regulatory protein P-II	*glnB*	3.23
A0A1Q5WH71	Thiol:disulfide interchange protein	*dsbA*	3.22
A0A0G8B466	2-dehydro-3-deoxygluconokinase	AR325_02155	3.22
A0A0M5K334	Transaldolase	*tal*	3.18
A0A080UWJ0	Peptidyl-prolyl cis-trans isomerase	*fklB*	3.15
A0A6N0D450	Two-component system response regulator BaeR	*baeR*	3.13
A0A6N0CZA0	Glucose-6-phosphate isomerase	*pgi*	3.10
A0A0F6KTS7	2-dehydro-3-deoxy-phosphogluconate aldolase	*eda*	3.10
V5YUY6	Stringent starvation protein A	*sspA*	3.09
A0A6M5HTX8	Uncharacterized protein	HMI62_14785	3.08
A0A1Q4NZT3	DUF1471 domain-containing protein	BHU62_12635	3.03
A0A086FJA0	50S ribosomal protein L9	*rplI*	3.02

**Table 4:** Identification of the downregulated proteins (<0.50-fold change) in FB1-treated *S. marcescens* 329-2 compared with the control group.

Entry	Protein Names	Gene Names	Fold Change
A0A514I8F1	DUF3251 domain-containing protein	FG174_04965	0.50
A0A086FBJ7	Outer membrane protein assembly factor BamD	*bamD*	0.48
A0A6G8TR73	Outer membrane lipoprotein RcsF	*rcsF*	0.48
A0A080UZC4	Tol-Pal system protein TolR	*tolR*	0.48
A0A2V4H3Z0	Ribose-phosphate pyrophosphokinase	*prs*	0.47
V5YU02	Divisome-associated lipoprotein YraP	*yraP*	0.47
A0A0M4S0F9	Glutamine synthetase	*glnA*	0.47
A0A379ZG33	Phage shock protein A	*pspA*	0.47
Q5J5B8	Outer membrane protein assembly factor BamC	*nlpbsm*	0.46
A0A379ZA39	Outer membrane protein slp	*slp*	0.46
A0A080V8M2	Heat shock chaperone IbpB	*ibpB*	0.46
A0A6I4HK57	Outer membrane protein assembly factor BamB	*bamB*	0.46
A0A0P0QBU0	Glycoprotein-polysaccharide metabolism protein	A8A12_17600	0.46
A0A6I6ZSG9	ATP synthase subunit alpha	*atpA*	0.46
A0A6N0CYE2	Phosphate acetyltransferase	*pta*	0.45
A0A6N0CUX7	Glycine-tRNA ligase subunit beta	*glyS*	0.43
A0A1Q5WD75	NAD(P)H dehydrogenase (quinone)	*wrbA*	0.42
A0A6I6ZPJ4	Terminase	GV243_19590	0.40
A0A3E2EPF1	Outer membrane	*ompA*	0.39
A0A1Q4NYR4	ATP synthase gamma chain	*atpG*	0.38
A0A6M5IGG0	SPOR domain-containing protein	HMI62_24000	0.35
A0A1Q4P2F3	Fructose-1,6-bisphosphatase class 1	*fbp*	0.34
A0A6H3SAN4	Glyceraldehyde-3-phosphate dehydrogenase	*gapA*	0.34
A0A0M3UJK8	ATP synthase subunit beta	*atpD*	0.30
A0A6G8TKN9	Uncharacterized protein	Uncharacterized protein	0.21

## Data Availability

Data are available upon request, please contact the contributing authors.

## Supplementary Materials

The following are available online at https://www.mdpi.com/article/10.3390/toxins13090638/s1, Table S1: Upregulation proteins in FB1-treated *S. marcescens* 329-2 compared with the control group, Table S2: Downregulation proteins in FB1-treated *S. marcescens* 329-2 compared with the control group.
